# TRAIL induces pro-apoptotic crosstalk between the TRAIL-receptor signaling pathway and TrkAIII in SH-SY5Y cells, unveiling a potential therapeutic “Achilles heel” for the TrkAIII oncoprotein in neuroblastoma

**DOI:** 10.18632/oncotarget.13098

**Published:** 2016-11-04

**Authors:** Luciana Gneo, Pierdomenico Ruggeri, Lucia Cappabianca, Antonietta Rosella Farina, Natalia Di Ianni, Andrew Reay Mackay

**Affiliations:** ^1^ Department of Applied Clinical and Biotechnological Sciences, University of L'Aquila, L'Aquila 67100, Italy

**Keywords:** TrkAIII oncoprotein, TRAIL, apoptosis, neuroblastoma, SH-SY5Y

## Abstract

TrkAIII expression in neuroblastoma (NB) associates with advanced stage disease, worse prognosis, post therapeutic relapse, and in NB models TrkAIII exhibits oncogenic activity and promotes chemotherapeutic-resistance. Here, we report a potential therapeutic “Achilles heel” for the TrkAIII oncoprotein in a SH-SY5Y NB model that is characterised by one-way TRAIL-induced, pro-apoptotic crosstalk between the TRAIL receptor signaling pathway and TrkAIII that results in the delayed induction of apoptosis. In TrkAIII SH-SY5Y cells, blocked in the intrinsic apoptosis pathway by elevated constitutive Bcl-2, Bcl-xL and Mcl-1 expression, TRAIL induced delayed caspase-dependent apoptosis via the extrinsic pathway and completely abrogated tumourigenic capacity *in vitro*. This effect was initiated by TRAIL-induced SHP-dependent c-Src activation, the induction of TrkAIII/SHP-1/c-Src complexing leading to SHP-mediated TrkAIII de-phosphorylation, subsequent induction of complexing between de-phosphorylated TrkAIII and cFLIP associated with a time-dependent increase the caspase-8 to cFLIP ratio at activated death receptors, resulting in delayed caspase cleavage and caspase-dependent apoptosis. We also confirm rate-limiting roles for c-FLIP and Mcl-1 in regulating the sensitivity of TrkAIII SH-SY5Y cells to TRAIL-induced apoptosis via the extrinsic and intrinsic pathways, respectively. Our study unveils a novel mechanism for the TRAIL-induced apoptosis of TrkAIII expressing NB cells that depends upon SHP/Src-mediated crosstalk between the TRAIL-receptor signaling pathway and TrkAIII, and supports a novel potential pro-apoptotic therapeutic use for TRAIL in TrkAIII expressing NB.

## INTRODUCTION

The developmental and stress-regulated alternative TrkAIII splice variant of the neurotrophin receptor tropomyosin related kinase A (TrkA) is expressed by advanced stage human neuroblastomas (NB) and NB cell lines, associates with metastatic disease, worse prognosis and post-therapeutic disease-relapse in high TrkA expressing unfavourable tumours and exhibits oncogenic activity in NB models [1–5]. It is characterised by exon 6–7 skipping and is expressed as an immature N-glycosylated 100 kDa protein that is devoid of the extracellular D4 Ig- like domain and several N-glycosylation sites that prevent spontaneous receptor activation and facilitate cell surface expression. In contrast to fully-spliced TrkA receptors, TrkAIII is miss-localised to intracellular membranes within which it undergoes ligand-independent activation that promotes self-perpetuating re-cycling between the endoplasmic reticulum (ER) and ERGIC and recruitment to the centrosome. The result is chronic oncogenic signaling through IP3K/Akt but not Ras/MAPK, a pro-survival ER-stress response characterised by ATF6 activation and increased Grp78/Bip expression, a more angiogenic and stem cell-like phenotype, centrosome amplification and genetic instability, increased resistance to ROS and chemotherapeutic agent-induced death and enhanced primary and metastatic tumour growth in NB models [1, 2, 6–12]. Although small molecular TrkA inhibitors and PNA inhibitors of TrkAIII expression do not directly induce the death of TrkAIII expressing NB cells, they reduce TrkAIII oncogenic activity and sensitize TrkAIII expressing NB cells to chemotherapeutic and cytotoxic agents [1, 12].

In pursuit of novel ways to kill NB cells, the pro-apoptotic TNFα family cytokine Apo2L/TRAIL has been identified as a promising chemotherapeutic candidate that acts selectively on tumour but not non-transformed cells [13–18]. TRAIL induces apoptosis by ligating functional DR4 and/or DR5 TRAIL receptors that recruit FADD and caspase 8 (or 10) to form the death-receptor complex, DISC, resulting in cleavage-dependent activation of caspase-8 (or –10), initiating apoptosis through the extrinsic pathway in type I tumour cells or via cBID to tBID cleavage through the intrinsic mitochondrial pathway in type 2 tumour cells [14, 19, 20]. In both tumour cell types, TRAIL-induced apoptosis is regulated by the equilibrium between functional (DR4 and DR5) and decoy receptors (DcR1, DcR2 and OPG), caspase 3, 8, 9 and 10 expression levels and the equilibrium between caspase 8 and its inhibitory analogues cFLIP_S_ and cFLIP_L_. In type I but not type 2 tumour cells, TRAIL-induced apoptosis is also regulated by the equilibrium between pro-apoptotic BH-3 only and anti-apoptotic B-cell lymphoma-2 (Bcl-2) proteins that regulate mitochondrial outer membrane permeability [14, 21–26]. Therapeutic TRAIL use in NB, however, has been hampered by reports of TRAIL-resistance [27–40], making the characterisation and circumvention of the mechanisms responsible fundamental for future therapeutic use of TRAIL in NB [41–48]. Within this context, we recently reported that NGF sensitizes TRAIL-resistant TrkA expressing NB cells to TRAIL-induced apoptosis, unveiling a novel immunological pro-apoptotic dimension to NGF/TrkA interaction and potential therapeutic use for NGF plus TRAIL in TrkA expressing NB [49, 50].

In the present study, we report a potential therapeutic “Achilles heel” for TrkAIII in a human SH-SY5Y NB model. We show that TRAIL induces one way, pro-apoptotic crosstalk between the TRAIL receptor signaling pathway and TrkAIII, resulting in the induction of delayed apoptosis through the extrinsic pathway and the complete abrogation of tumourigenic activity *in vitro*. This TRAIL-induced pro-apoptotic cross talk is mediated by SHP protein tyrosine phosphatase(s) and the non-receptor tyrosine kinase c-Src, depends upon TrkAIII de-phosphorylation, and TrkAIII binding and sequester of cFLIP, which increases the caspase-8 to cFLIP ratio at activated death receptors, inducing delayed caspase-dependent apoptosis.

## RESULTS

### TrkAIII sensitizes SH-SY5Y cells to TRAIL-induced apoptosis

**Figure 1:**
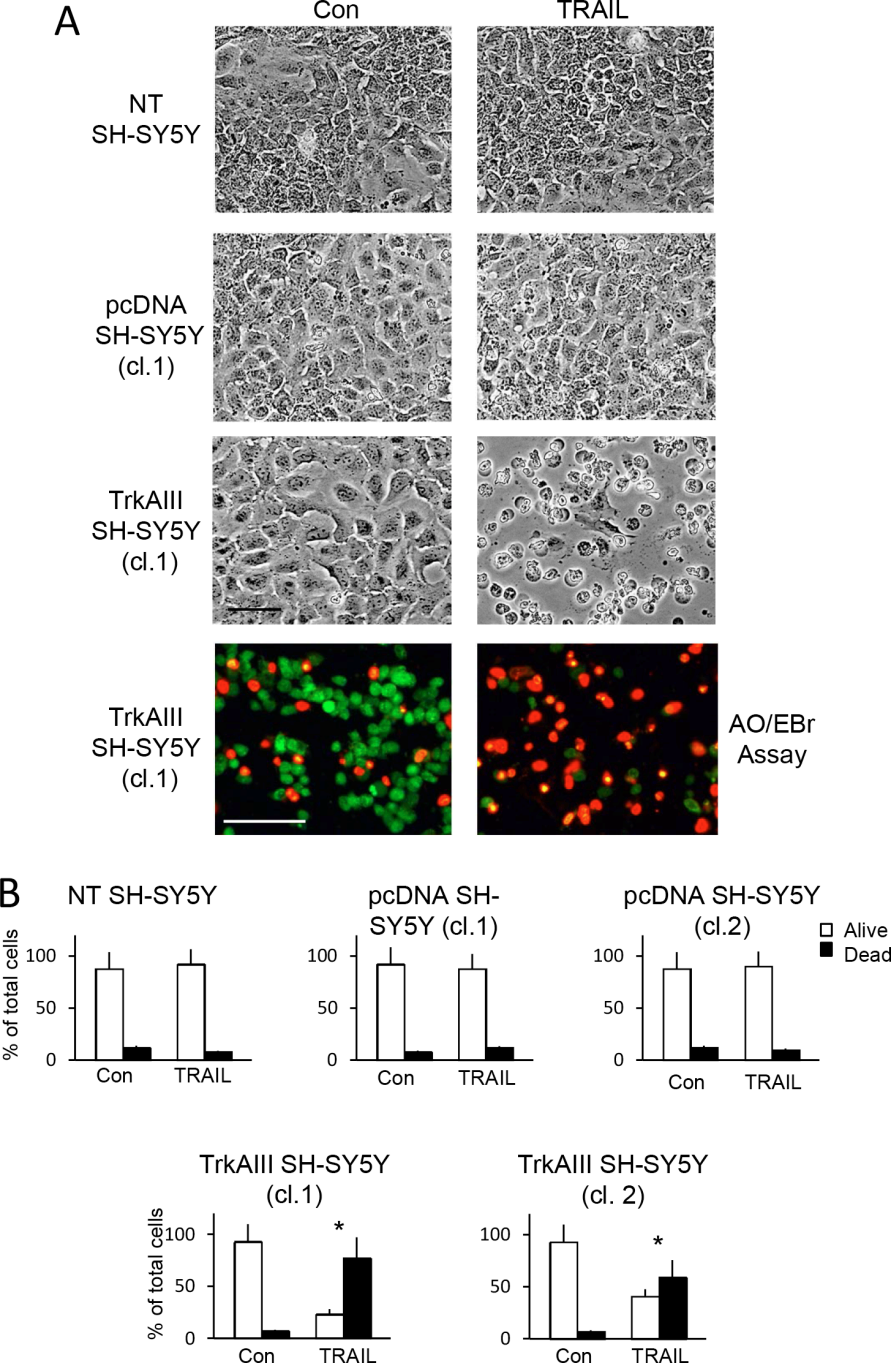
TRAIL induces apoptosis of TrkAIII SH-SY5Y cells (**A**) Representative phase contrast (black and white) and fluorescent (green and orange) micrographs (Bar = 100 μm) demonstrating marked induction of TrkAIII SH-SY5Y but not non-transfected (NT) SH-SY5Y or pcDNA SH-SY5Y cell death, following 24 hours incubation with TRAIL (200 ng/ml). (**B**) Histograms displaying the mean (± SD) percentage (%) survival (white) and death (black) cells of NT SH-SY5Y, independent pcDNA SH-SY5Y clones (cl.1 and 2) and independent TrkAIII SH-SY5Y clones (cl.1 and cl.2) incubated for 24 hours with TRAIL (200 ng/ml), in three independent cell death assays each performed in duplicate (* = statistical significance with respect to Con).

In 2-dimensional cultures, TRAIL at concentrations > 50 ng/ml induced significant cell death of two independent TrkAIII expressing SH-SY5Y cell lines (clones 1 and 2) but not two independent empty pcDNA3.1 vector (pcDNA) transfected SH-SY5Y cell lines (clones 1 and 2) or non-transfected (NT) parental SH-SY5Y cells (Figure 1A and 1B, data shown for TRAIL concentration of 200 ng/ml only). TRAIL-induced cell death was confirmed by Acridine Orange/ethidium bromide cell death assay (Figure 1A) and caspase-dependent apoptosis was confirmed by the complete inhibition of TRAIL-induced death by z-VAD-fmk pan caspase and z-IETD-fmk caspase 8 inhibitors (see Figure 8). Maximal apoptosis occurred at TRAIL concentrations > 100 ng/ml, and at 200 ng/ml TRAIL induced a mean (±SD) of 77.2 ± 26.2% (*P* = 0.0002, *n* = 6) death in TrkAIII SH-SY5Y clone 1 and 59.3 ± 28.4% (*p* = 0.0026, *n* = 6) death in TrkAIII SH-SY5Y clone 2, measured at 16 hours (Figure 1B).

**Figure 2:**
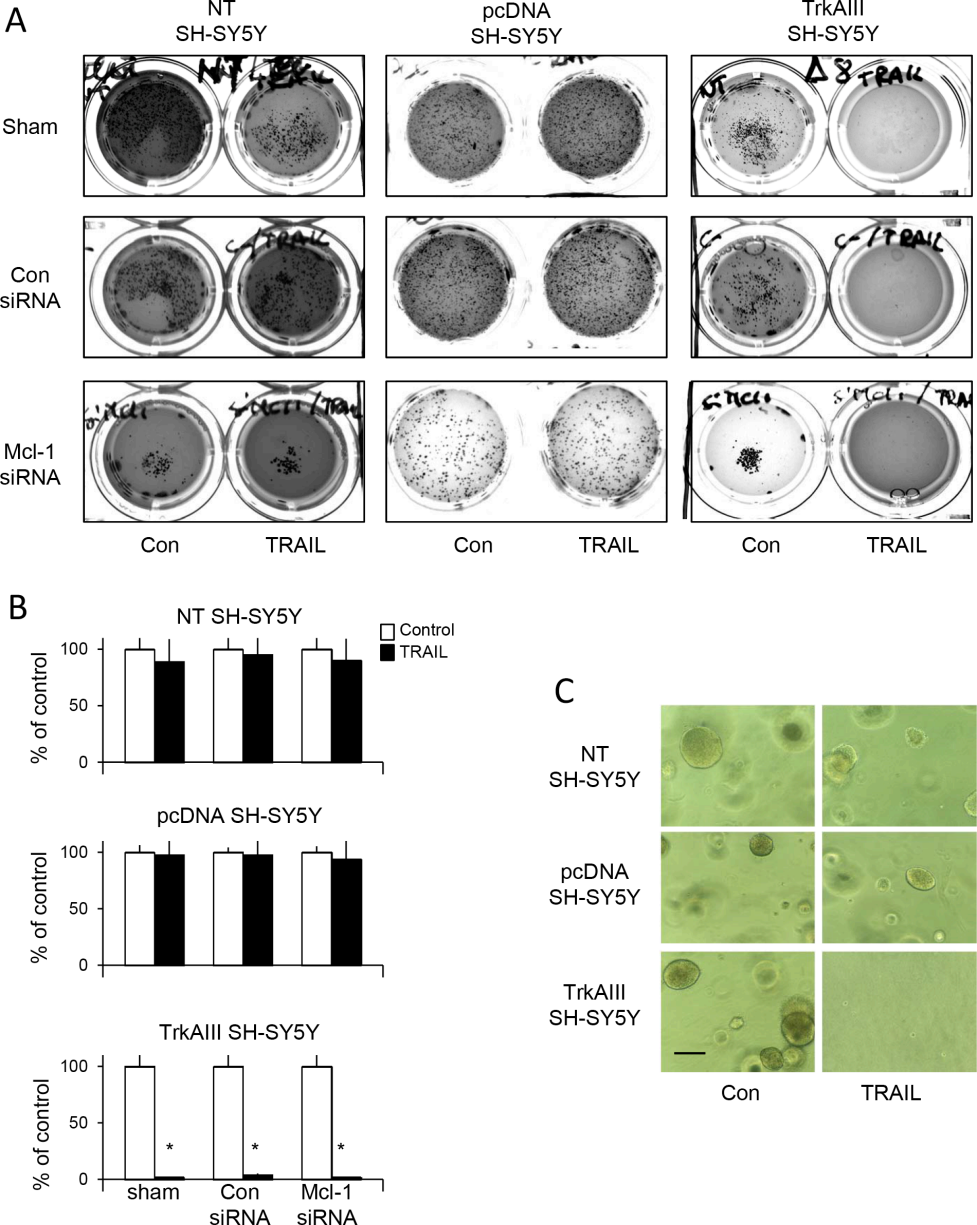
TRAIL abrogates the tumorigenic activity of TrkAIII SH-SY5Y cells *in vitro* (**A**) Representative photographs, demonstrating the abrogation of TrkAIII SH-SY5Y but not NT SH-SY5Y or pcDNA SH-SY5Y tumourigenic activity *in vitro,* in the presence but not absence of TRAIL (200 ng/m). (**B**) Histograms demonstrating the mean (±SD) percentage change in tumour numbers grown from NT SH-SY5Y, pcDNA SH-SY5Y and TrkAIII SH-SY5Y cells, in the absence (100%) or presence of TRAIL (200 ng/ml). Tumour sphere numbers were evaluated in 10 ×10 magnification fields in triplicate experiments, each performed in duplicate (* = statistical significance compared to untreated control). (**C**) Representative phase contrast micrographs demonstrating the appearance of tumour spheroid grown from NT SH-SY5Y, pcDNA SH-SY5Y and TrkAIII SH-SY5Y in the absence (con) or presence of TRAIL (200 ng/ml) (bar = 1 mm).

In substrate-independent tumourigenesis assays *in vitro,* TRAIL (200 ng/ml) completely abrogated tumourigenic growth of TrkAIII SH-SY5Y clone 1 and 2 but did not reduce the tumourigenic growth of either pcDNA SH-SY5Y clone 1 and clone 2 or NT SH-SY5Y cells over 14 days (Figure 2A–2C, data displayed for NT SH-SY5Y, pcDNA SH-SY5Y clone 1 and TrkAIII SH-SY5Y clone 1, only). SiRNA knockdown of Mcl-1 in NT-SH-SY5Y or pcDNA SH-SY5Y cells did not reduce tumorigenic activity *in vitro* in the presence of TRAIL (200 ng/ml) over 14 days (Figure 2A) nor sensitize to NT-SH-SY5Y or pcDNA SH-SY5Y cells to TRAIL-induced apoptosis (not shown). The clear difference in NT-SH-SY5Y, pcDNA SH-SY5Y and TrkAIII SH-SY5Y tumourigenic activity *in vitro* in the presence of TRAIL (200 ng/ml) is demonstrated in Figure 2C, at higher magnification. TrkAIII SH-SY5Y clone 1 and pcDNA SH-SY5Y clone 1 were selected for further study.

Together, these data show that TrkAIII sensitizes SH-SY5Y cells to TRAIL-induced apoptosis, resulting in the abrogation of tumorigenic activity *in vitro*.

### NT, pcDNA and TrkAIII SH-SY5Y cells express all components required for TRAIL-induced apoptosis

**Figure 3:**
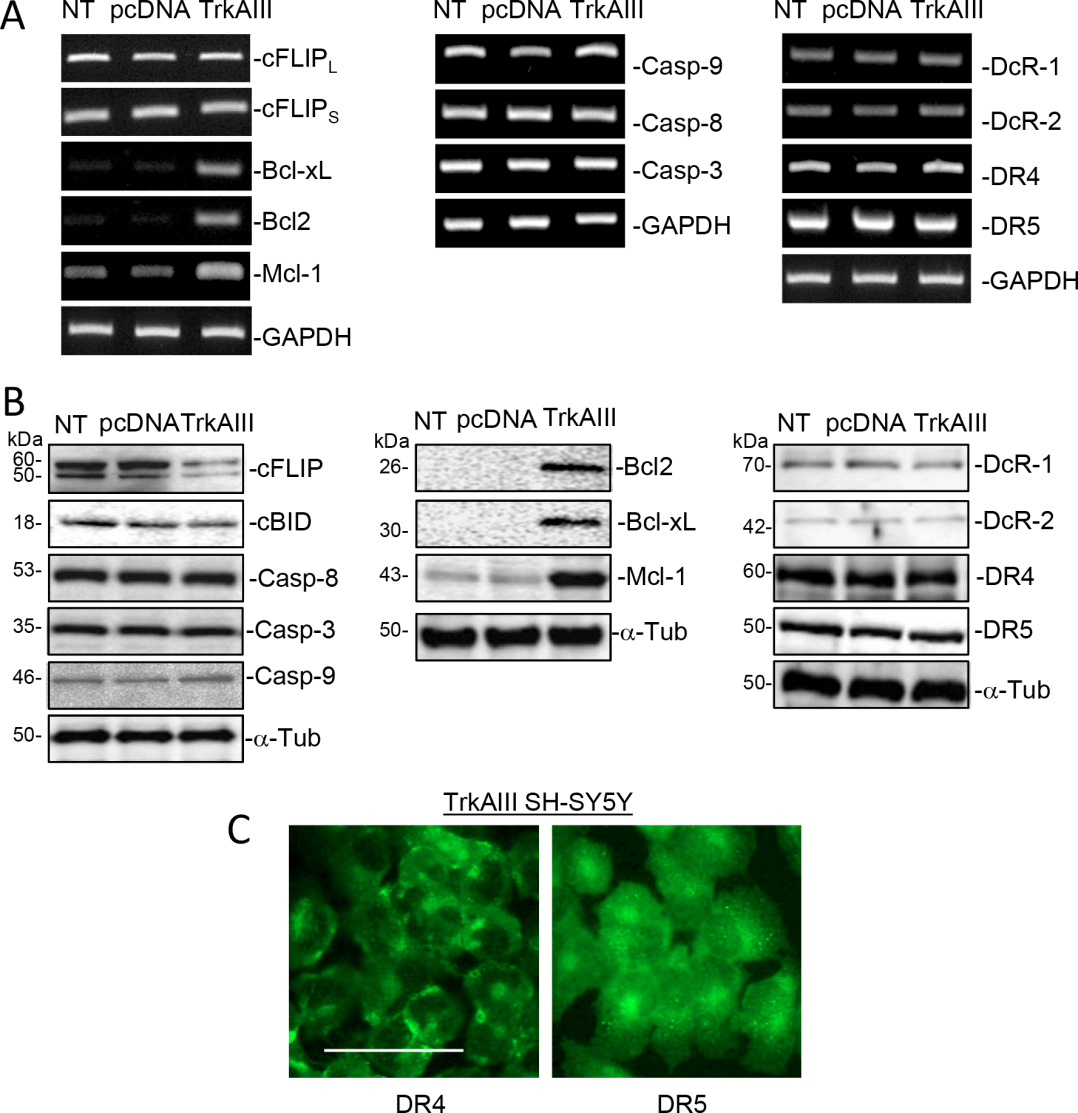
NT, pcDNA and TrkAIII SH-SY5Y cells express all of the major components required for TRAIL-induced apoptosis (**A**) Ethidium bromide stained agarose gels demonstrating similar levels of RT-PCR products for major components involved in TRAIL-induced apoptosis generated from RNAs purified from NT-SH-SY5Y, pcDNA SH-SY5Y and TrkAIII SH-SY5Y cells. (**B**) Western blots demonstrating similar protein levels of major components involved in TRAIL-induced apoptosis in whole NT-SH-SY5Y, pcDNA SH-SY5Y and TrkAIII SH-SY5Y cell extracts (20 μg). (**C**) Representative IF micrographs demonstrating cell surface expression of functional DR4 and DR5 TRAIL receptor in non-permeabilized TrkAIII SH-SY5Y cells (bar = 100 μm).

Western blot and RT-PCR comparisons of NT SH-SY5Y, pcDNA SH-SY5Y and TrkAIII SH-SY5Y cells revealed similar levels of functional (DR4 and DR5) and decoy (DcR1 and DcR2) TRAIL receptor mRNA and protein expression (Figure 3A and 3B), with cell surface DR4 and DR5 expression detected in TrkAIII SH-SY5Y cells by indirect IF of non-permeabilized cells (Figure 3C), adding to our previous report of cell surface DR4 and DR5 expression in NT SH-SY5Y and pcDNA SH-SY5Y cells [49]. NT, pcDNA and TrkAIII SH-SY5Y cell lines also exhibited similar levels of constitutive caspase-3, caspase-8, caspase-9 mRNA and protein expression and cBID protein expression (Figure 3A and 3B). In contrast to NT and pcDNA SH-SY5Y cells, TrkAIII SH-SY5Y cells also exhibited higher constitutive mRNA and protein expression of the intrinsic apoptosis pathway inhibitors Bcl- 2, Bcl-xL and Mcl-1, and exhibited lower levels of cFLIP protein but not mRNA expression (Figure 3A and 3B).

**Figure 4:**
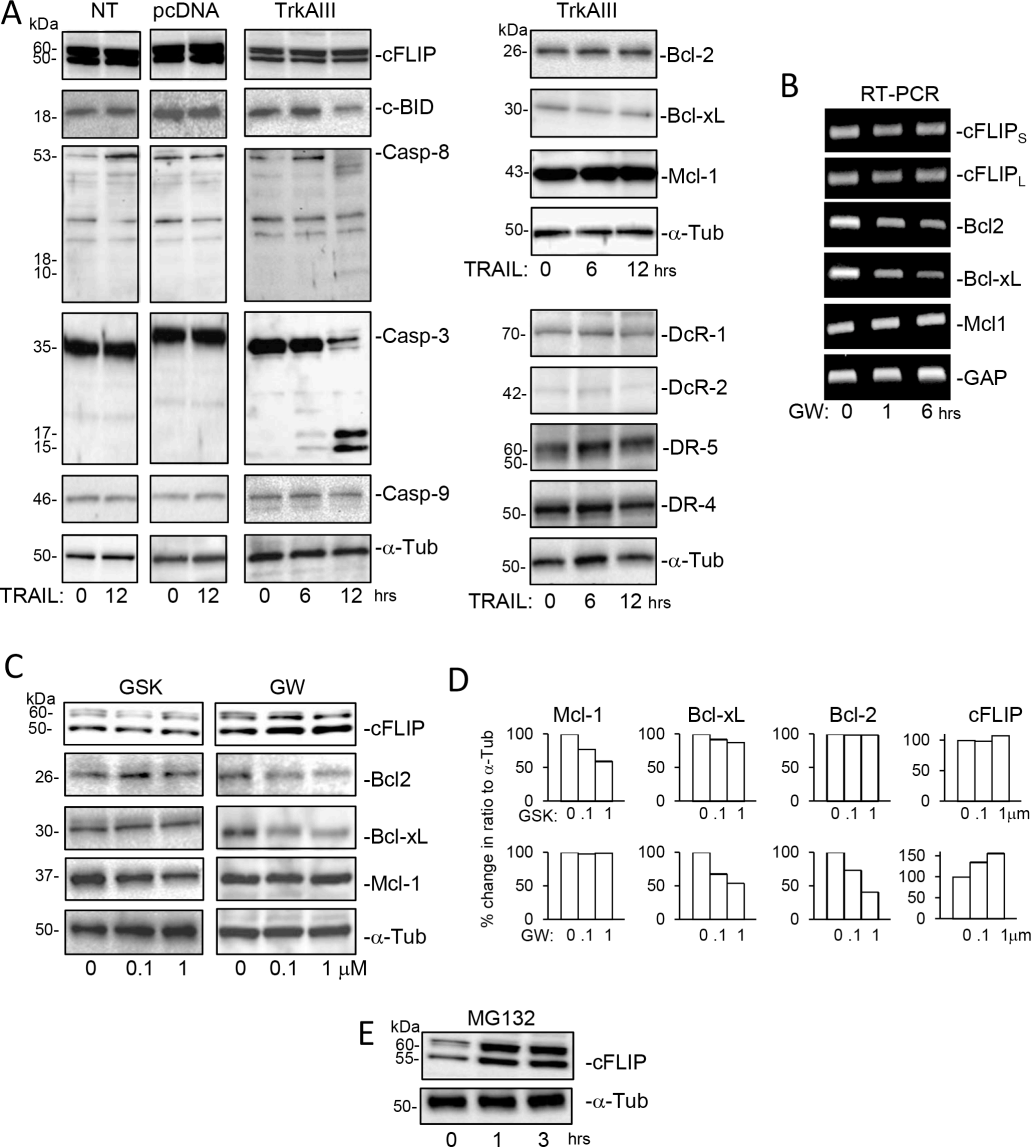
Effects of TRAIL, GW441756, GSK2656157 and MG132 upon the expression of components of TRAIL-induced apoptosis (**A**) Western blots demonstrating the effect of TRAIL (200 ng/ml 0–12 hours) upon cFLIP, cBID, Caspase-8, Caspase-3 and Caspase-9 levels in whole cell extracts from NT SH-SY5Y (NT), pcDNA SH-SY5Y (pcDNA) and TrkAIII SH-SY5Y cells (TrkAIII) and Bcl-2, Bcl-xL, Mcl-1, DcR-1, DcR-2, DR-5 and DR-4 protein levels in whole cell extracts from TrkAIII SH-SY5Y cells (TrkAIII) (20 μg of whole cell extract loaded per sample). (**B**) Agarose gels demonstrating cFLIP_S_, cFLIP_L_, Bcl-2, Bcl-xL, Mcl-1 and GAPDH RT-PCR product levels generated from mRNAs purified from TrkAIII SH-SY5Y cells treated with 1 μM GW441756 (GW; 0, 1 and 6 hours). (**C**) Western blots demonstrating the inhibitory effect of the PERK inhibitor GSK2656157 (GSK; 0.1 and 1 μM for 24 hours) upon Mcl-1 but not cFLIP, Bcl-2, Bcl-xL or α-tubulin (α-Tub) expression and the inhibitory effect of the TrkA inhibitor GW441756 (GW: 0.1 and 1.0 μM for 24 hours) upon Bcl-2 and Bcl-xL but not Mcl-1 or α-tubulin expression and its stimulatory effect upon cFLIP expression in whole cell TrkAIII SH-SY5Y cell extracts (20 μg). (**D**) Histograms of the adjacent Western blots, displaying the results described in (C), expressed as percentage change in densitometric ratio to α-tubulin. (**E**) Western blots demonstrating the effects of MG132 (10 μM, 0–3 hours) upon cFLIP and α-tubulin protein levels in whole cell extracts from TrkAIII SH-SY5Y cells (20 μg).

Treatment of TrkAIII SH-SY5Y cells with TRAIL (200 ng/ml for 0–12 hours) did not alter the levels of DR4, DR5, DcR1, DcR2, Bcl-2a, Bcl-xL, Mcl-1 or cFLIP protein expression but did induced delayed (post 6 hours) cleavage of caspase-3 and caspase-8, and also a delayed reduction in c-BID protein levels, consistent with degradation, not detected in NT-SH-SY5Y or pcDNA SH- SY5Y cells (Figure 4A). Treatment of TrkAIII SH-SY5Y cells with the TrkA inhibitor GW441756 (0.1 and 1 μM for 24 hours) [51] decreased Bcl-2 and Bcl-xL mRNA and protein expression, increased cFLIP protein but not mRNA expression (Figure 4B and 4C) but did not alter Mcl-1 mRNA or protein expression (Figure 4B and 4C). In contrast, treatment of TrkAIII SH-SY5Y cells with the PERK inhibitor GSK2656157 (0.1 and 1 μM) reduced Mcl-1 but not Bcl-2 or Bcl-xL protein expression and did not modulate cFLIP protein levels (Figure 4B and 4C).

The possibility that degradation at the proteasome was responsible for reducing cFLIP protein levels in TrkAIII SH-SY5Y cells was confirmed using the proteasome inhibitor MG132 (10 μM), which increased cFLIP protein levels in TrkAIII SH-SY5Y cells (Figure 4E).

Together, these data show that resistance to TRAIL-induced apoptosis does not result from the lack of TRAIL-induced apoptosis component expression or the expression of Bcl2 family inhibitors but is associated with higher levels of cFLIP expression. Conversely, TrkAIII SH-SY5Y sensitivity to TRAIL-induced apoptosis is associated with reduced cFLIP levels that result from TrkAIII activity and cFLIP degradation at the proteasome and occurs despite increased Bcl2 family expression.

### TRAIL-induced apoptosis is delayed, caspase-dependent and cFLIP and Mcl-1-regulated

**Figure 5:**
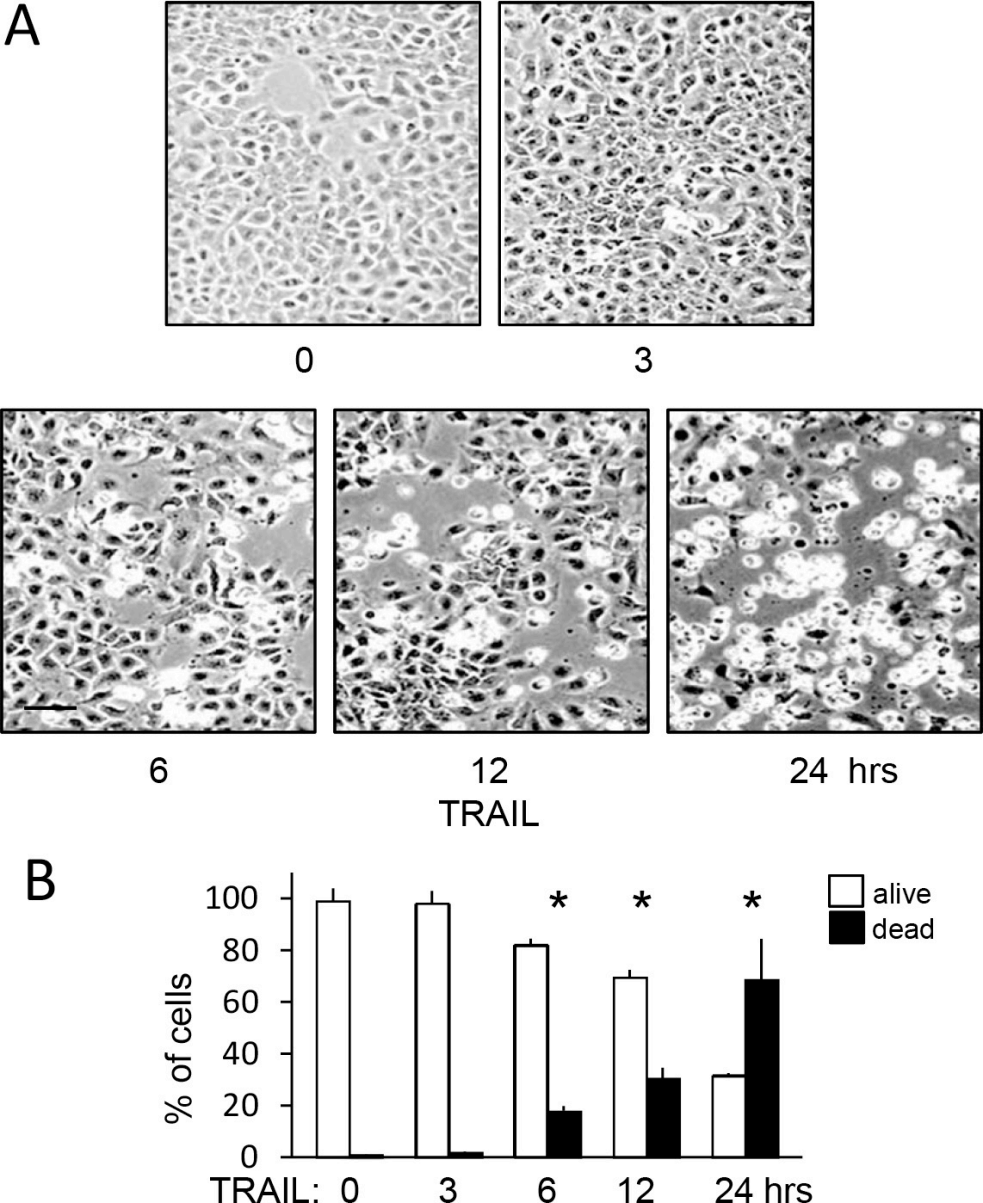
TRAIL induces delayed and not immediate apoptosis of TrkAIII SH-SY5Y cells (**A**) Representative phase (bar = 100 μm) contrast micrographs demonstrating time-dependent TRAIL-induced (200 ng/ml) TrkAIII SH-SY5Y cell death from 0–24 hours. (**B**) Histogram demonstrating the mean (± SD) percentage survival (white) and death (black) of TrkAIII SH-SY5Y cells incubated for 0, 3, 6, 12 and 24 hours with TRAIL (200 ng/ml) in three independent AO/EBr cell death assays, each performed in duplicate (* = statistical significance compared to TRAIL 0 hr control).

TRAIL (200 ng/ml)-induced TrkAIII SH-SY5Y apoptosis was not detected prior to 6 hours. At 6 hours, TRAIL-induced a mean (±SD) of 18.3 ± 9.8% cell death (*p* = 0.0264, *n* = 6), 30.4 ± 13.8% cell death at 12 hours (*p* = 0.0033, *n* = 6) and 68.4 ± 23.4% cell death at 24 hours (*p* < 0.0001, *n* = 6) (Figure 5A and 5B).

**Figure 6:**
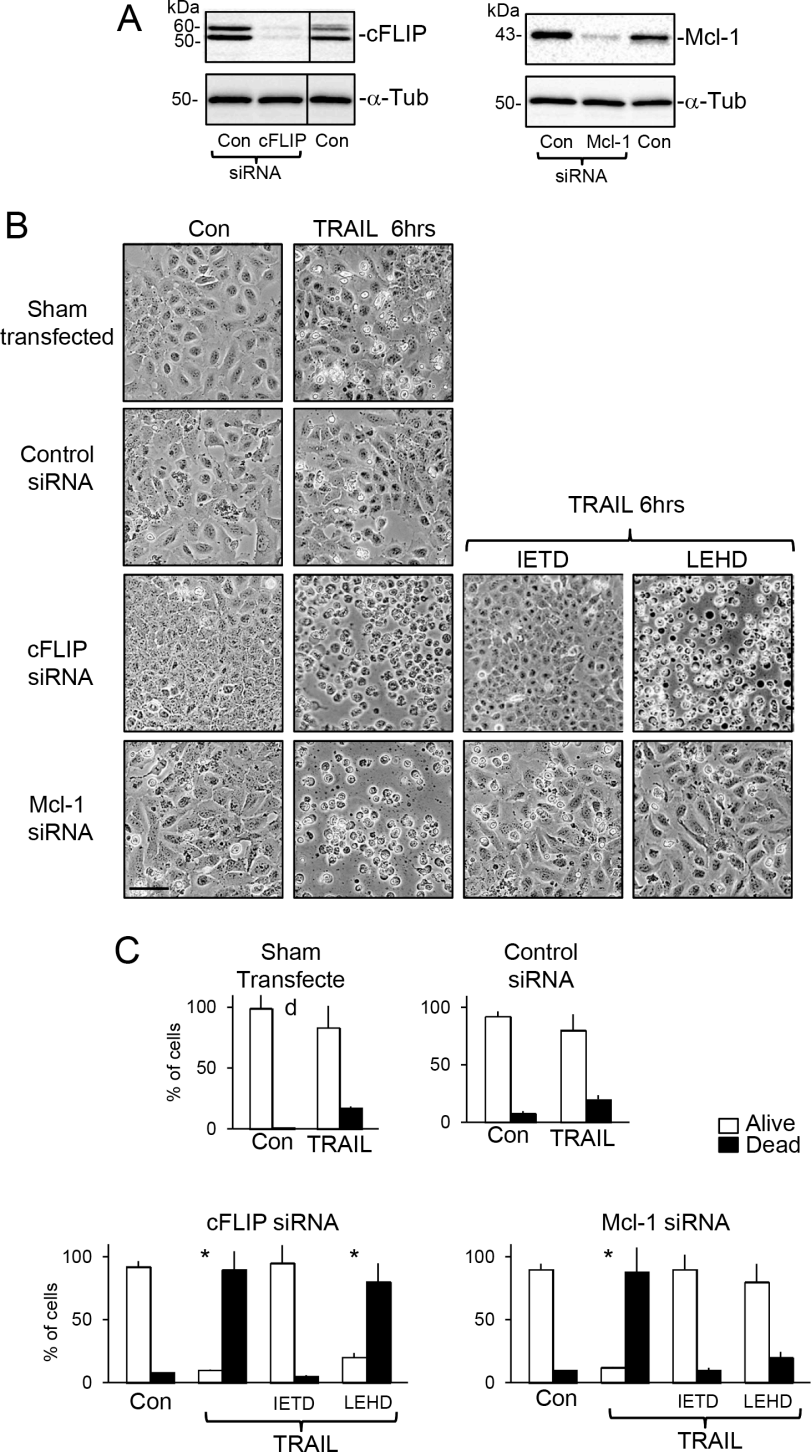
SiRNA cFLIP and Mcl-1 knockdown accelerate TRAIL-induced apoptosis (**A**) Western blots demonstrating knockdown of cFLIP and Mcl-1 protein expression in whole cell extracts from TrkAIII SH-SY5Y cells transiently transfected with cFLIP- and Mcl-1-specific siRNAs but not control siRNAs, compared to cFLIP and Mcl-1 levels in sham-treated controls (Con). (**B**) Representative phase contrast micrographs demonstrating acceleration of TRAIL-induced (200 ng/ml) death at 6 hours of TrkAIII SH-SY5Y cells exhibiting siRNA cFLIP and Mcl-1 knockdown, plus the inhibitory effect of z-IETD-fmk (IETD: 10 μM) but not z-LEHD-fmk (LEHD: 10 μM) on TRAIL-induced apoptosis of TrkAIII SH-SY5Y cells exhibiting siRNA cFLIP knockdown plus the inhibitory effects of both z-IETD-fmk and z-LEHD-fmk on TRAIL-induced apoptosis of TrkAIII SH-SY5Y cells exhibiting siRNA Mcl-1 knockdown, at 6 hours. (bar = 100 μm). (**C**) Histograms showing the mean (±SD) percentage of survival (white) and death (black) of sham (transfection reagent alone), control siRNA-transfected TrkAIII SH-SY5Y cells and TrkAIII SH-SY5Y cells exhibiting siRNA-induced cFLIP and Mcl-1 knockdown, following 6 hour TRAIL-treatment (200 ng/ml), in the presence or absence (Con) of z-IETD-fmk (IETD: 10 μM) or z-LEHD-fmk (LEHD: 10 μM), in 3 independent cell death assays, each performed in duplicate (* = significance difference compared to control).

**Figure 7:**
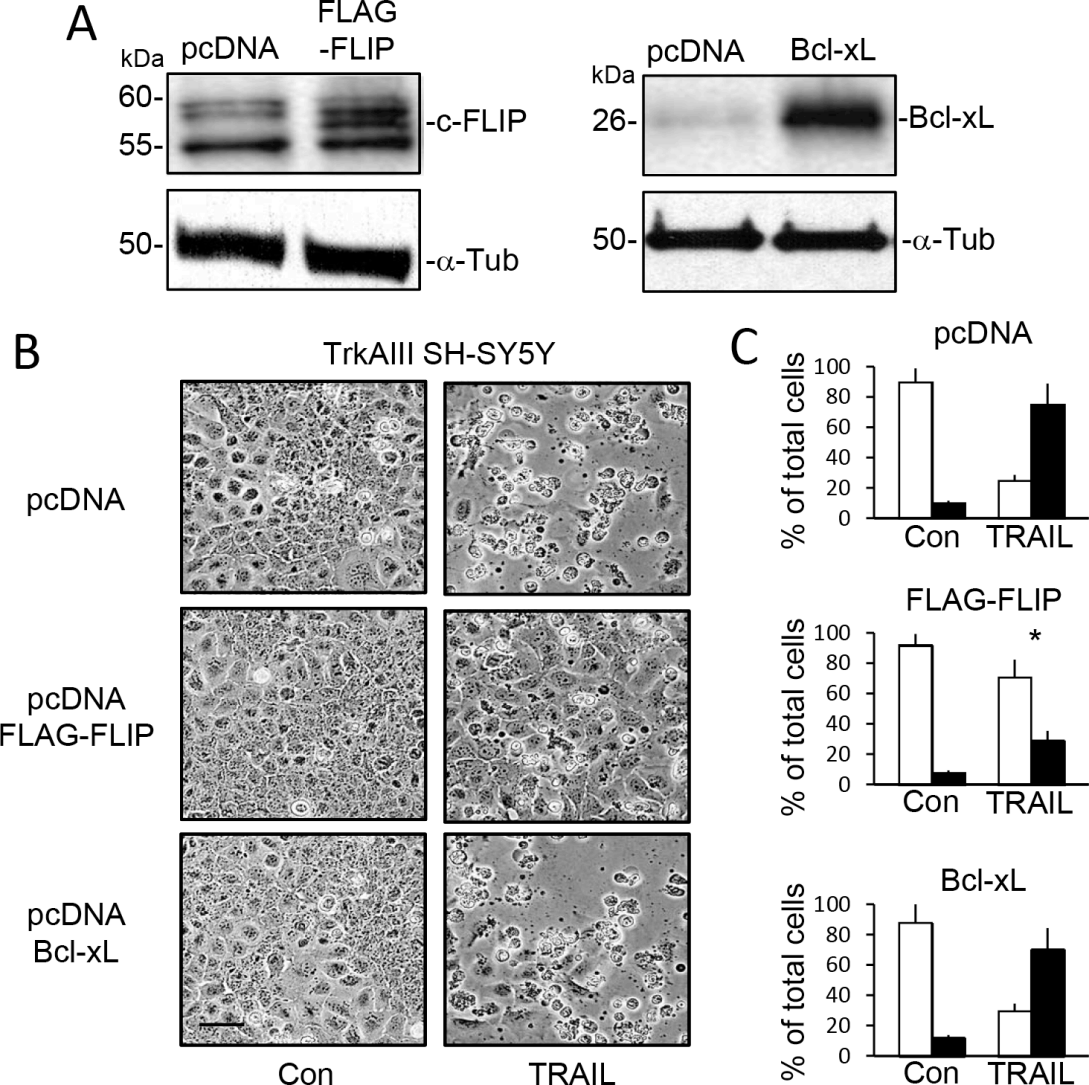
Overexpression of cFLIP but not Bcl-xL inhibits TRAIL-induced TrkAIII SH-SY5Y cell apoptosis (**A**) Western blots demonstrating expression of exogenous FLAG-cFLIP and Bcl-xL in TrkAIII SH-SY5Y transient transfectants compared to empty pcDNA transfected counterparts (pcDNA). (**B**) Representative phase contrast micrographs demonstrating reduced levels of TRAIL-induced (200 ng/ml for 24 hours) apoptosis in TrkAIII cells overexpressing FLAG-cFLIP compared control pcDNA transfected TrkAIII SH-SY5Y cells and TrkAIII SH-SY5Y cells overexpressing Bcl-xL (bar = 100 μm). (**C**) Histograms displaying the mean (± SD) percentage (%) of survival (white) or death (black) of TrkAIII SH-SY5Y cells transfected with empty pcDNA vector (pcDNA), overexpressing exogenous FLAG-cFLIP or overexpress exogenous Bcl-xL, following TRAIL-treatment (200 ng/ml for 24 hours) in three independent AO/EBr cell death assays, each performed in duplicate (* = significant difference compared to empty pcDNA transfected TrkAIII SH-SY5Y control (pcDNA)).

SiRNA cFLIP knockdown in TrkAIII SH-SY5Y cells (Figure 6A) significantly accelerated and augmented TRAIL (200 ng/ml)-induced apoptosis to a mean (±SD) of 90.7 ± 15.8% at 6 hours (*P* < 0.0001, *n* = 4), compared to 17.2 ± 8.6% in sham-transfected and 20.3 ± 18.6% in control siRNA transfected TrkAIII SH-SY5Y counterparts (Figure 6B and 6C). SiRNA Mcl-1 knockdown in TrkAIII SH-SY5Y cells (Figure 6A) also significantly accelerated and augmented TRAIL (200 ng/ml)-induced death to 88.5 ± 22.2% at 6 hours (*P* < 0.0001, *n* = 4), compared to both sham and control siRNA transfected counterparts (Figure 6B and 6C). TRAIL-induced death of TrkAIII SH-SY5Y cells exhibiting cFLIP knockdown was completely abrogated by z-IETD-fmk caspase-8 inhibitor (10 μM) but not by z-LEHD-fmk (10 μM) caspase-9 inhibitor (*p* < 0.0001, *n* = 4), whereas both z-IETD-fmk (10 μM) and z-LEHD-fmk (10 μM) significantly inhibited TRAIL-induced death of TrkAIII SH-SY5Y cells exhibiting Mcl-1 knockdown (*p* < 0.0001, *n* = 4 for both) (Figure 6C). Transient FLAG-tagged cFLIP overexpression in TrkAIII SH-SY5Y cells (Figure 7A) significantly reduced TRAIL (200 ng/ml)-induced apoptosis at 24 hours from a mean (± SD) of 75.3 ± 18.2% in TrkAIII SH-SY5Y cells transfected with empty pcDNA vector to 29.2 ± 18.4% (*P* < 0.02, *n* = 4) (Figure 7B and 7C). Transient Bcl-xL overexpression (Figure 7A) did not significantly inhibit TRAIL-induced TrkAIII SH-SY5Y apoptosis compared to empty pcDNA vector-transfected TrkAIII SH-SY5Y counterparts (Figure 7B and 7C).

**Figure 8:**
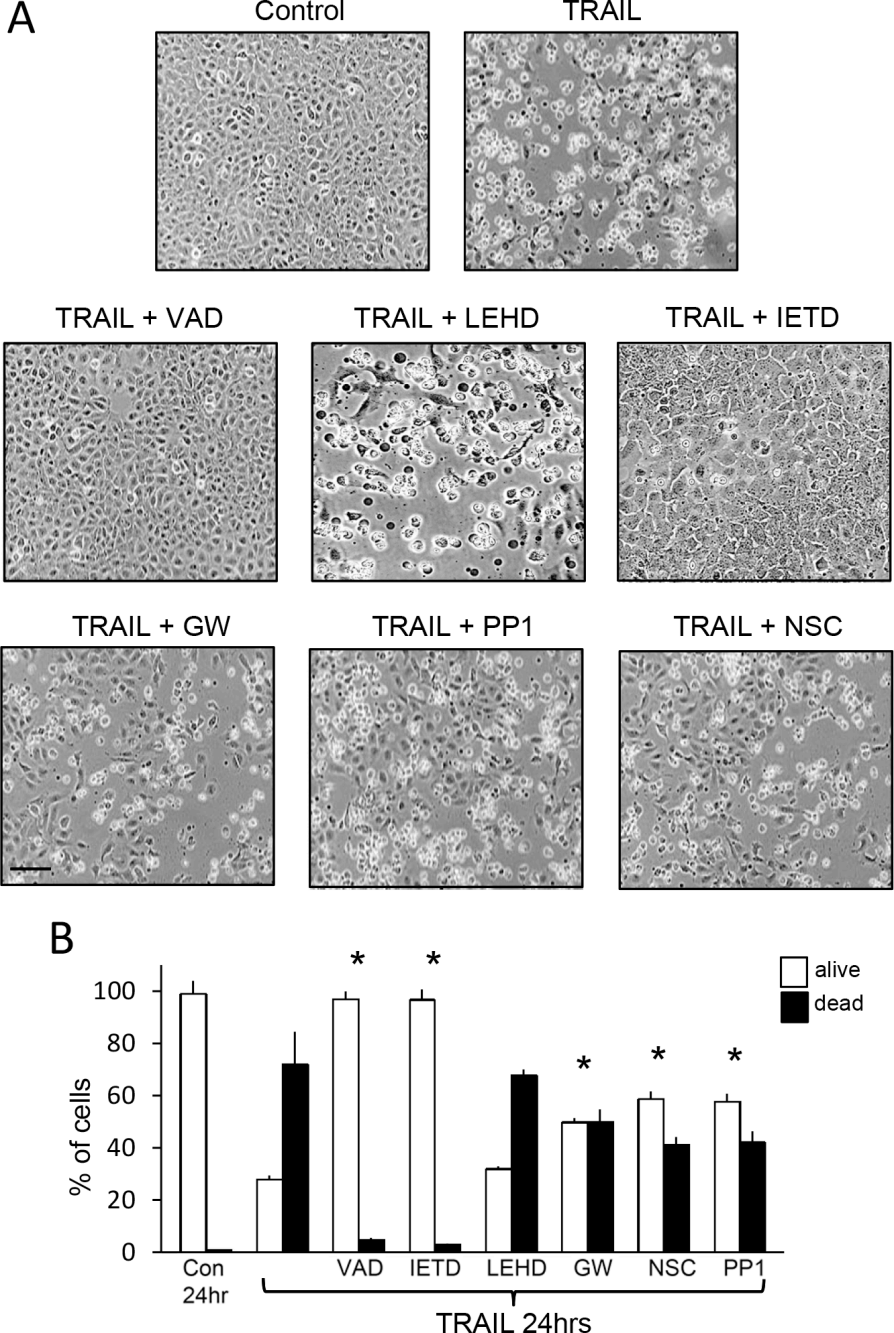
TRAIL-induced TrkAIII SH-SY5Y apoptosis is inhibited by z-VAD-fmk, z-IETD-fmk, GW441756, PP1 and NSC-87877 but not by z-LEHD-fmk (**A**) Representative phase contrast micrographs demonstrating inhibition of TRAIL-induced TrkAIII SH-SY5Y apoptosis following 24 hour treatment (200 ng/ml) by 10 μM z-VAD-fmk (VAD), 10 μM z-IETD-fmk (IETD) but not 10 μM z-LEHD-fmk (LEHD) and by 1 μM GW441756 (GW), 1 μM PP1 and 1 μM NSC-87877 (NSC) (bar = 100 μm). (**B**) Histograms displaying the mean (+SD) percentage (%) TrkAIII SH-SY5Y survival (white) or death (black) following treatment with TRAIL alone (200 ng/ml), TRAIL plus 10 μM z-VAD-fmk (VAD), TRAIL plus 10 μM z-LEHD-fmk (LEHD), TRAIL plus 10 μM z-IETD-fmk (IETD), TRAIL plus 1 μM GW441756 (GW), TRAIL plus 1 μM PP1 and TRAIL plus 1 μM NSC-87877 (NSC), in three independent AO/EBr experiments, each performed in duplicate (* = significant difference compared to TrkAIII SH-SY5Y cells treated for 24 hours with TRAIL in the absence of inhibitors).

Under non-knockdown conditions, TRAIL (200 ng/ml)-induced TrkAIII SH-SY5Y apoptosis was abrogated by z-IETD-fmk (10 μM) caspase-8 inhibitor and by z-VAD-fmk (10 μM) pan-caspase inhibitor but not by z-LEHD-fmk (10 μM) caspase-9 inhibitor (Figure 8A and 8B).

Together, these data confirm that TRAIL-induced TrkAIII SH-SY5Y apoptosis is caspase-dependent, delayed in association with delayed caspase activation (see Figure 4A), is regulated by cFLIP and occurs via the extrinsic pathway. Furthermore, the data identify Mcl-1 as the dominant inhibitor of the intrinsic apoptosis pathway in TrkAIII SH-SY5Y cells and show that reducing cFLIP or Mcl-1 expression augments sensitivity to TRAIL-induced apoptosis.

### TRAIL activates c-Src, induces TrkAIII complexing with c-Src and SHP-1 and TRAIL-induced apoptosis involves TrkAIII, c-Src and SHP activity

Mean (±SD) TRAIL-induced TrkAIII SH-SY5Y death of 72.2 ± 17.3% at 24 hours was significantly reduced to 50.1 ± 9.3% (*P* = 0.0203, *n* = 6) by GW441756 (1 μM) TrkA inhibitor (51); to 41.3 ± 7.2% (*P* = 0.0023, *n* = 6) by NSC87877 (1 μM) SHP-1/2 inhibitor (52) and to 42.2 ± 9.8% (*P* = 0.0041, *n* = 6) by PP1 (1 μM) c-Src inhibitor (53) (Figure 8A and 8B).

**Figure 9:**
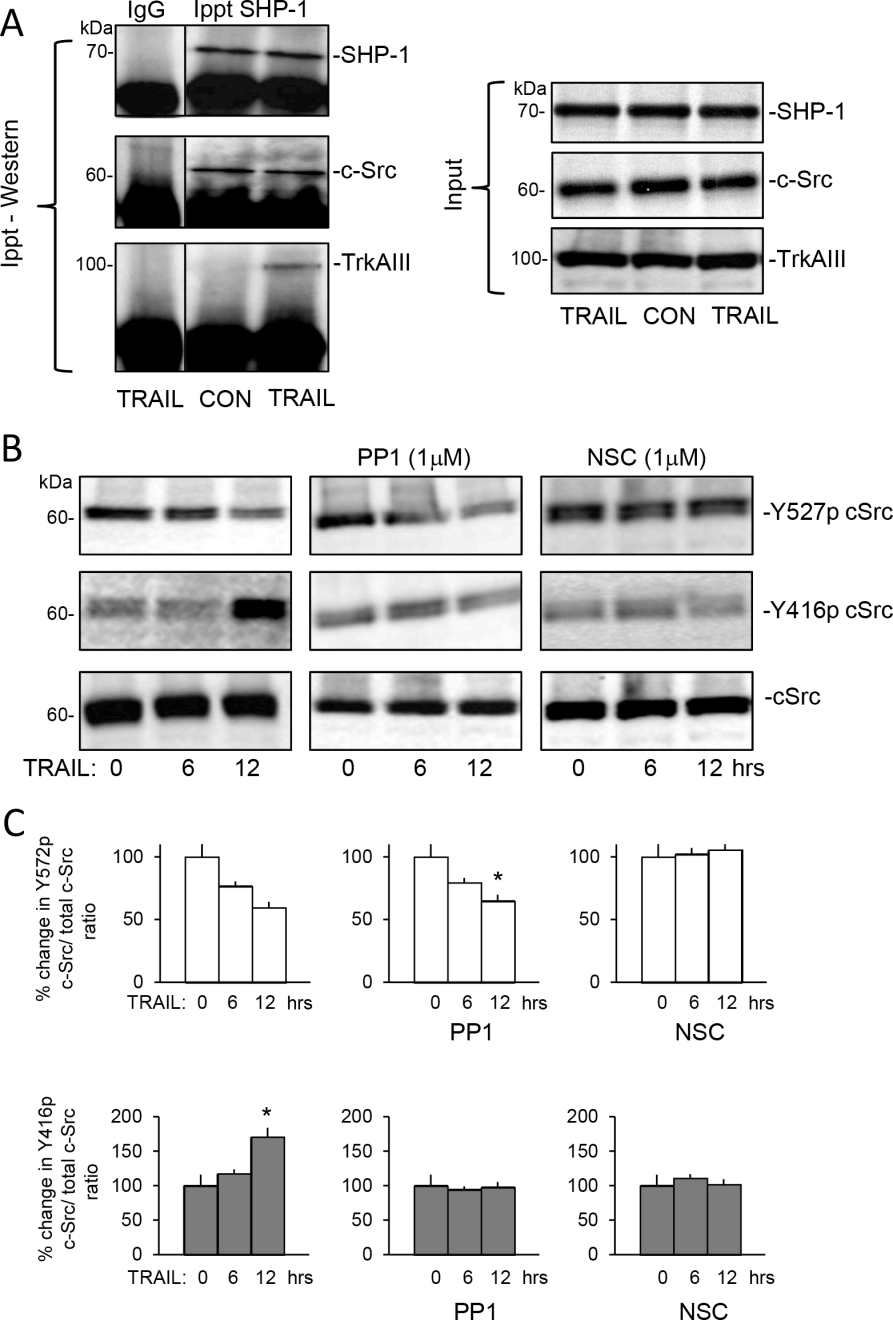
TRAIL induces complexing between TrkAIII, SHP-1 and c-Src and activates c-Src in TrkAIII SH-SY5Y cells (**A**) Immunoprecipitation/Western blots demonstrating SHP-1 pull down of c-Src but not TrkAIII from untreated TrkAIII SH-SY5Y whole cell extracts (500 μg) and SHP-1 pull down of both c-Src and TrkAIII in TRAIL-treated (200 ng/ml for 12 hours) TrkAIII SH-SY5Y whole cell extracts (500 μg), plus Western blots showing input levels of SHP-1, c-Src and TrkAIII in TrkAIII SH-SY5Y whole cell extracts (20 μg). (**B**) Western blots demonstrating TRAIL-induced (200 ng/ml for 0, 6 and 12 hours) c-Src Y527 de-phosphorylation and stimulation of c-Src Y416 phosphorylation; 1 μM PP1 inhibition of TRAIL-induced stimulation of c-Src Y416 phosphorylation but not TRAIL-induced c-Src Y527 de-phosphorylation, and 1 μM NSC-87877 (NSC) inhibition of both TRAIL-induced stimulation of c-Src Y416 phosphorylation and c-Src Y527 de-phosphorylation, compared to total c-Src levels in whole cell TrkAIII SH-SY5Y extracts (20 μg). (**C**) Histograms demonstrating the mean (+SD) change in densitometric ratio of total to Y527-phosphorylated c-Src and total to Y416-phosphorylated c-Src associated with TrkAIII SH-SY5Y cells treated with TRAIL (200 ng/ml) alone, TRAIL plus 1 μM PP1 or TRAIL plus 1 μM NSC-87877 (NSC) for 0, 6 and 12 hours, assessed by ImageJ densitometry of 3 independent Western blots of whole TrkAIII SH-SY5Y extracts (20 μg) (* = significant difference compared to untreated (TRAIL 0 hrs controls).

SHP-1 immunoprecipitated from untreated whole TrkAIII SH-SY5Y cell extracts (500 μg) pulled down c-Src but not TrkAIII, confirming constitutive SHP-1/c-Src but not SHP-1/TrkAIII complexing (Figure 9A). TRAIL (200 ng/ml for 6 hours) did not alter SHP-1/c-Src complexing but induced SHP-1 pull-down of TrkAIII together with c-Src (Figure 9A).

Western blots detected high constitutive c-Src Y527 phosphorylation relative to Y416 phosphorylation in TrkAIII SH-SY5Y cells (Figure 9B). TRAIL (200 ng/ml, 0–12 hours) reduced c-Src Y527 phosphorylation at 6 and 12 hours in association with increased c-Src Y416 phosphorylation, consistent with c-Src activation (Figure 9B). PP1 (1 μM) c-Src inhibitor did not alter either constitutive c-Src Y527 or Y416 phosphorylation or TRAIL-induced c-Src Y527 de-phosphorylation in TrkAIII SH-SY5Y cells over the 12 hour time course but did abrogate TRAIL stimulation of c-Src Y416 phosphorylation, consistent with autophosphorylation (Figure 9B and 9C). NSC87877 (1 μM) SHP1/2 inhibitor prevented both TRAIL-induced Src Y527 de- phosphorylation and increased Y416 phosphorylation (Figure 9B and 9C), implicating SHP in TRAIL-induced c-Src activation via the de-phosphorylation of Y527.

**Figure 10:**
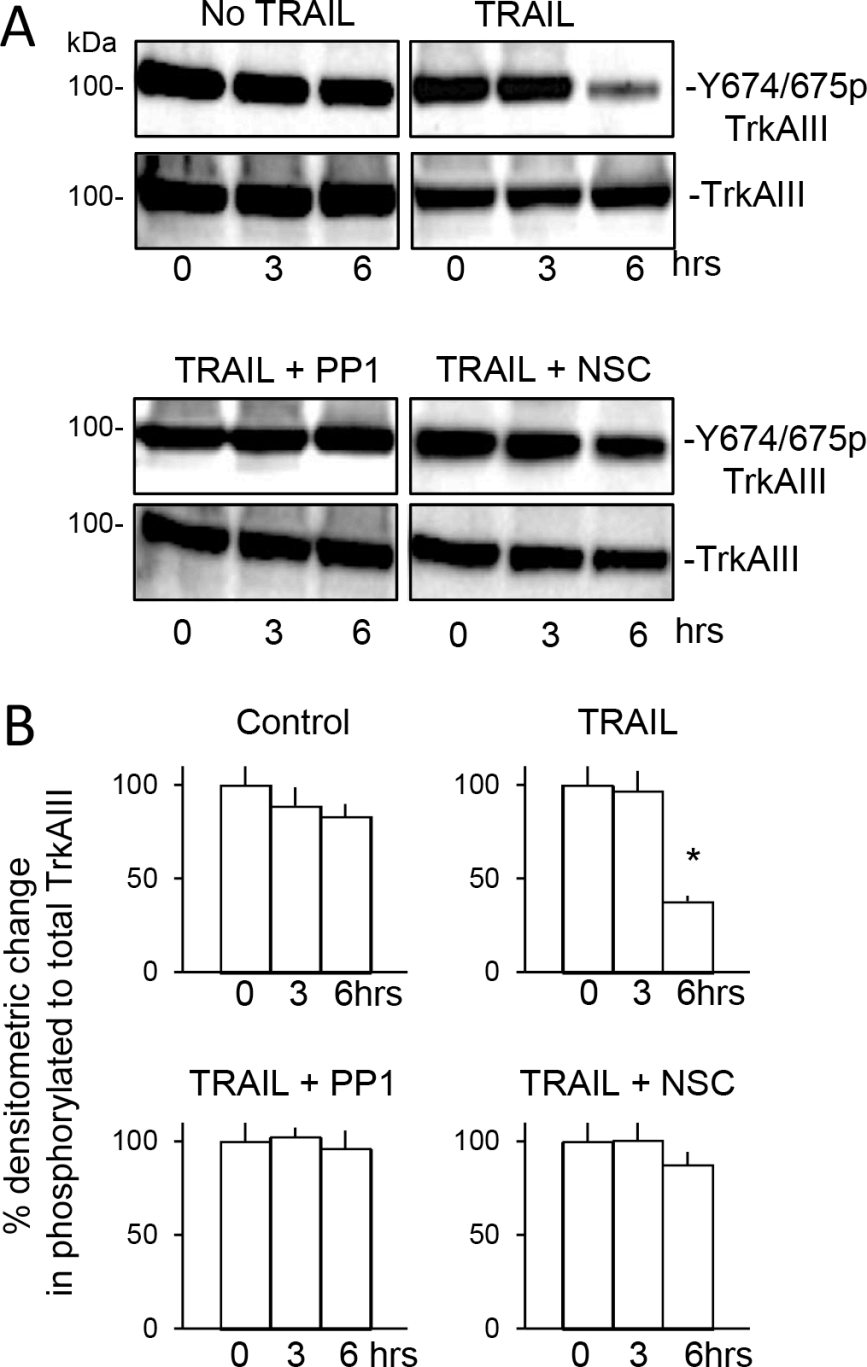
TRAIL-induced de-phosphorylation of TrkAIII Y674/675 associates with apoptosis and is prevented by PP1 and NSC-87877 (**A**) Western blots demonstrating no change total and Y674/675 phosphorylated TrkAIII levels in TrkAIII SH-SY5Y cells incubated for 0, 3 and 6 hours in absence of TRAIL, compared to time-dependent TRAIL-induced (200 ng/ml) TrkAIII Y674/675 de-phosphorylation associated with constant TrkAIII levels, in TrkAIII SH-SY5Y cells incubated for 3 and 6 hours plus the inhibition of TRAIL-induced TrkAIII Y674/675 de-phosphorylation by 1 μM PP1 (TRAIL + PPI) and by 1 μM NSC-87877 (TRAIL + NSC) at both 3 and 6 hours. (**B**) Histograms demonstrating the mean (±SD) change the ratio of total to Y674/675-phosphorylated TrkAIII in whole cell extracts (20 μg) from TrkAIII SH-SY5Y cells incubated for 0–6 hours in the absence of TRAIL (control) in the presence of TRAIL (200 ng/ml) alone (TRAIL), with TRAIL plus 1 μM PP1 (TRAIL + PP1) or with TRAIL plus 1 μM NSC-87877 (TRAIL + NSC), assessed by ImageJ densitometry of 3 independent Western blots (* = significant difference compared to untreated 6 hour controls).

**Figure 11:**
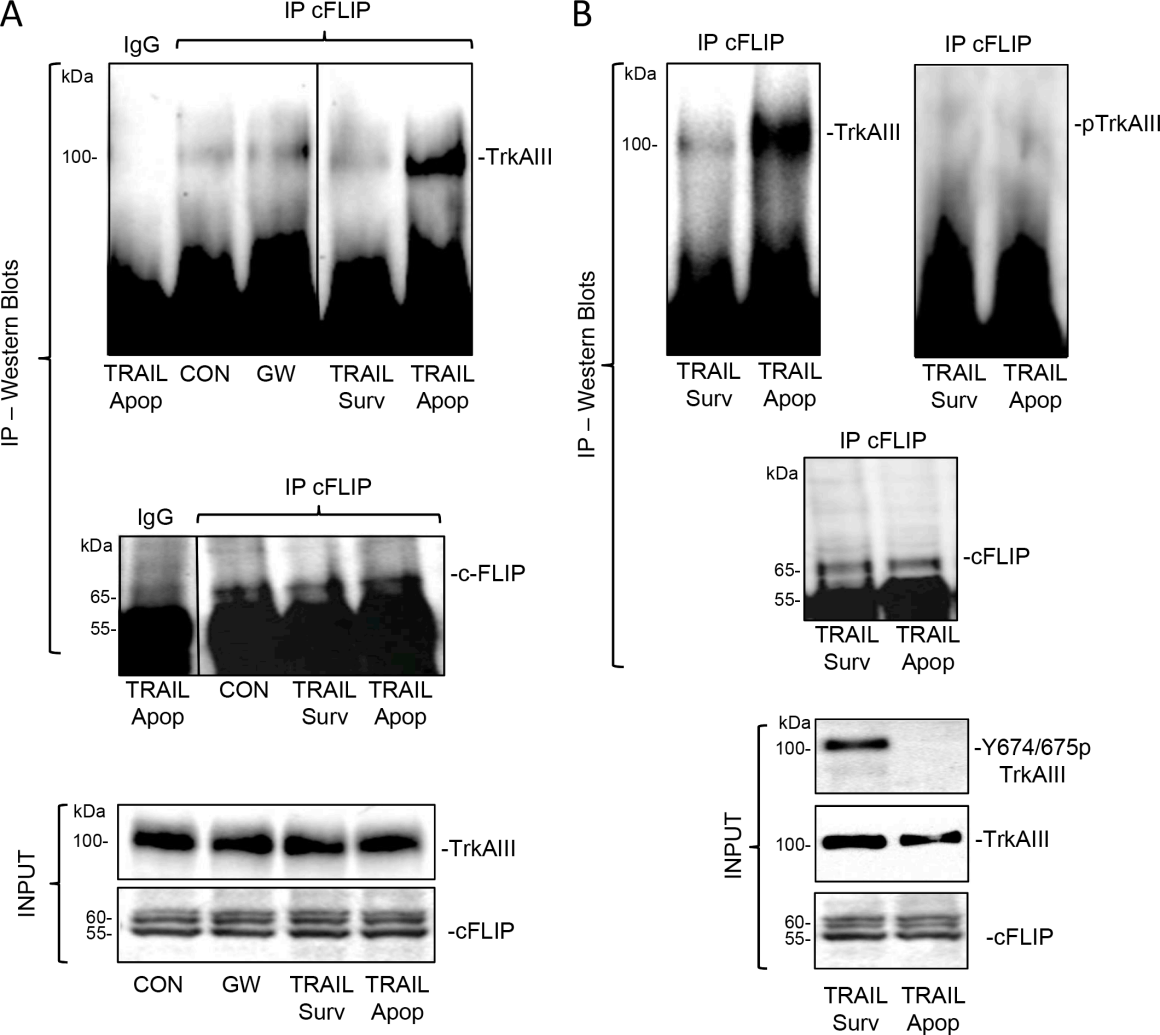
TRAIL induced complexing between Y674/675 de-phosphorylated TrkAIII and endogenous cFLIP associates with apoptotis (**A**) Immunoprecipitation/Western blots demonstrating cFLIP pull down of TrkAIII in whole cell extracts (500 μg) from untreated (CON), GW441756-treated (GW: 1 μM for 24 hours) and TRAIL-treated (200 ng/ml for 12 hours) apoptotic (Apop) and surviving (Surv) TrkAIII SH-SY5Y cells. Pre-immune IgG controls are provided (IgG) and input levels of TrkAIII and cFLIP shown by regular Western blot (INPUT). (**B**) Immunoprecipitation/Western blots demonstrating cFLIP pull down of non-phosphorylated TrkAIII (Upper left and right panels) in whole cell extracts (500 μg) from TRAIL-treated (200 ng/ml for 12 hours) apoptotic (Apop) but not surviving (Surv) TrkAIII SH-SY5Y cells. Input levels of Y674/675 phosphorylated TrkAIII, total TrkAIII and cFLIP in TRAIL-treated apoptotic and surviving whole TrkAIII SH-SY5Y cell extracts are also displayed (INPUT).

In addition to inducing TrkAIII/SHP-1/c-Src complexing, TRAIL also induced de-phosphorylation of TrkAIII Y674/675 within 6 hours (Figure 10A and 10B), prevented by both PP1 and NSC87877 (Figure 10A and 10B) in association with significant inhibition of TRAIL-induced apoptosis, confirming roles for both c-Src and SHP in TRAIL-induced TrkAIII de-phosphorylation and subsequent apoptosis. PP1 and NSC87877 did not reduce constitutive TrkAIII Y674/675 phosphorylation in the absence of TRAIL (data not shown). Association between TRAIL-induced TrkAIII Y674/675 de-phosphorylation and apoptosis was confirmed in Western blots by exclusive detection of TrkAIII Y674/675 de-phosphorylation in apoptotic but not surviving TrkAIII SH-SY5Y cells (see Figure 11B, lower panel).

Together, these data implicate both SHP and c-Src in TRAIL-induced pro-apoptotic crosstalk with TrkAIII, and implicate SHP in c-Src activation and TrkAIII de-phosphorylation.

### TRAIL promotes TrkAIII/cFLIP complexing and alters the caspase-8 to cFLIP ratio recruited to death receptor complexes

In co-immunoprecipitation experiments, cFLIP immunoprecipitated from untreated whole TrkAIII SH-SY5Y cell extracts pulled down very low levels of TrkAIII, that were not reduced further by GW441756 (1 μM for 24 hours) (Figure 11A). C-FLIP immunoprecipitates pulled down non-phosphorylated TrkAIII from TRAIL-treated (200 ng/ml for 24 hours) apoptotic but not surviving TrkAIII SH-SY5Y cells (Figure 11A and 11B). TRAIL-induced TrkAIII Y674/675 de-phosphorylation was confirmed in input samples from apoptotic but not surviving cells (Figure 11B, INPUT).

**Figure 12:**
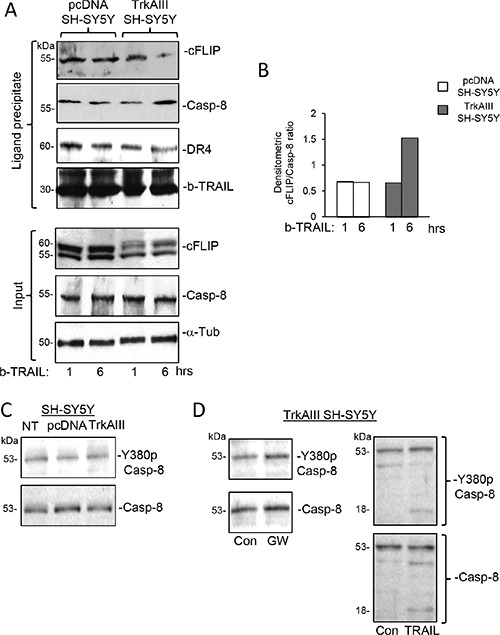
TRAIL induces a time-dependent increase in the caspase-8 to cFLIP ratio at ligand-activated death receptors in TrkAIII SH-SY5Y but not pcDNA SH-SY5Y cells (**A**) Western blots demonstrating reduced cFLIP levels and increased caspase-8 levels in ligand-precipitated DR4 positive death receptor complexes from TrkAIII SH-SY5Y but not pcDNA SH-SY5Y cells treated with biotin-labelled TRAIL (500 ng/ml) for 6 hours compared to 1 hour. Input levels of cFLIP, Caspase 8 (Casp-8) and α-tubulin (α-Tub) in total cell extracts (20 μg) are provided (Input). (**B**) Histogram displaying differences in the c-FLIP to caspase-8 ratios in the adjacent Western blots, adjusted for differences in DR4 densitometric levels, in ligand-precipitated DR4 positive death receptors purified from pcDNA SH-SY5Y (white) and TrkAIII SH-SY5Y cells (grey), obtained by image J densitometry. (**C**) Western blots demonstrating similar levels of total and Y380 phosphorylated caspase 8 in NT SH-SY5Y, pcDNA SH SY5Y and TrkAIII SH-SY5Y whole cell extracts (20 μg). (**D**) Western blots demonstrating no reduction in the levels of Y380 phosphorylated caspase 8 in whole cell extracts (20 μg) from TrkAIII SH-SY5Y treated in the presence or absence of GW441756 (GW, 1 μM for 24 hours) or TRAIL (200 ng/ml for 12 hours), plus TRAIL-induced caspase 8 cleavage to an 18kDa Y380 phosphorylated fragment.

In ligand-precipitation/Western blots, streptavidin precipitation of biotin-labelled TRAIL-ligated DR4 positive death receptor complexes from TrkAIII SH-SY5Y cells treated for 1 and 6 hours with biotinylated TRAIL, revealed an increase in the caspase 8 to cFLIP ratio from 0.66 following 1 hour TRAIL-treatment to 1.53 following 6 hour TRAIL-treatment. In contrast, the caspase-8 to cFLIP ratio of 0.68 pcDNA SH-SY5Y cells treated for 1 hour with biotinylated TRAIL remained at the same level in 6 hour-treated cells (Figure 12A and 12B).

Together, these data link TRAIL-induced SHP/c-Src-mediated TrkAIII de-phosphorylation and complexing with cFLIP to a pro-apoptotic increase in caspase-8 to cFLIP ratio at TRAIL-activated death receptors.

### TRAIL does not modulate Caspase-8 Y387 phosphorylation in TrkAIII SH-SY5Y cells

Western blots detected similar constitutive levels of inhibitory caspase 8 Y380 phosphorylation [54], in NT SH-SY5Y, pcDNA SH-SY5Y and TrkAIII SH-SY5Y cells. Neither GW441756 (1 μM for 24 hours) nor TRAIL (200 ng/ml for 12 hours) reduced Caspase 8 Y380 phosphorylation levels in TrkAIII SH-SY5Y cells (Figure 12C and 12D). These data associate constitutive inhibitory caspase-8 Y380 phosphorylation with the TRAIL-resistant SH-SY5Y phenotype, show that caspase-8 Y380 phosphorylation in TrkAIII SH-SY5Y cells is not TrkAIII-dependent and confirm that caspase-8 Y380 de-phosphorylation is not involved in sensitizing TrkAIII SH-SY5Y cells to TRAIL-induced apoptosis.

## DISCUSSION

In the present study, we report a potential therapeutic “Achilles heel” for the TrkAIII oncoprotein in a SH-SY5Y NB model. In this model, TRAIL induced delayed caspase-dependent apoptosis of SH-SY5Y cells engineered to express TrkAIII, resulting in the complete abrogation of tumorigenic growth capacity *in vitro*. Consistent with the TRAIL-resistant phenotype of parental and control-transfected SH-SY5Y cells (this study and 49), TRAIL-induced apoptosis of TrkAIII SH-SY5Y cells resulted from one-way SHP/c-Src-mediated pro-apoptotic crosstalk between the TRAIL receptor signaling pathway and TrkAIII. This results from SHP/c-Src-dependent binding and de-phosphorylation of TrkAIII, inducing cFLIP complexing with de-phosphorylated TrkAIII, increasing the caspase-8 to cFLIP ratio at TRAIL-activated death receptors, which explains the delayed induction of caspase cleavage and apoptosis, with rate-limiting roles confirmed for both c-FLIP and Mcl-1.

All the cell lines used in this study exhibited constitutive expression of components required for TRAIL-induced apoptosis (this study and 49), a pro-apoptotic expression equilibrium between functional and decoy TRAIL receptors and cell-surface functional DR4 and DR5 TRAIL receptor expression (this study and 49). In contrast to some NB cell lines [55], all of our cell lines expressed caspase 8 mRNA and protein. However, inhibitory caspase-8 Y380 phosphorylation [54] was also detect in all cell lines, providing an additional mechanism for reducing SH-SY5Y sensitivity to TRAIL-induced apoptosis (this study and 49).

TrkAIII SH-SY5Y cells expressed lower levels of cFLIP but higher levels of Bcl-2, Bcl-xL and Mcl-1 than either NT SH-SY5Y or pcDNA SH-SY5Y cells. Inhibitor (GW441756 and MG132) studies indicated that lower cFLIP protein expression by TrkAIII SH-SY5Y cells was post-transcriptional and dependent upon enhanced cFLIP degradation at the proteasome and TrkAIII tyrosine kinase activity. We are currently investigating whether this may depend upon Nedd-4 family E3 ligases, which bind activated TrkA receptors and promote cFLIP degradation at the proteasome [56, 57].

TRAIL-induced TrkAIII SH-SY5Y apoptosis was caspase-dependent and was abrogated by z-VAD-fmk pan-caspase and z-IETD-fmk caspase-8 inhibitors but not by z-LEHD-fmk caspase-9 inhibitor [58–60] or Bcl-xL overexpression. This characterises TrkAIII SH-SY5Y cells as type I tumour cells, sensitive to TRAIL-induced apoptosis through the extrinsic pathway [14, 19, 20, 25], with the intrinsic pathway blocked by constitutive Bcl-2, Bcl-xL and Mcl-1 expression (this study and 49).

TRAIL-induced TrkAIII SH-SY5Y apoptosis was delayed and not immediate, was not detected prior to 6 hours and increased to a maximum post 12 hours. Delayed apoptosis was associated with delayed cleavage of caspase 8 and delayed cleavage of the caspase 8 substrates c-BID [61] and caspase 3 [62], confirming a delay in caspase 8 activation. Therefore, low cFLIP levels combined with inhibitory caspase 8 Y380 phosphorylation were sufficient to prevent immediate apoptosis, implicating additional TRAIL-induced time-dependent pro apoptotic changes.

The central role of cFLIP in delaying TRAIL-induced TrkAIII SH-SY5Y apoptosis was confirmed by siRNA knockdown, which accelerated and augmented apoptosis to > 90% within 6 hours, and by cFLIP over expression that significantly inhibited TRAIL-induced TrkAIII SH-SY5Y apoptosis. This suggests that TRAIL-induced reduction in cFLIP function resulting from pro-apoptotic crosstalk with TrkAIII may underpin the delay in TRAIL-induced apoptosis. However, since TRAIL did not reduce cFLIP protein expression in TrkAIII SH-SY5Y cells any further reduction in cFLIP function would have to be post-translational.

Inhibitor studies confirmed that TRAIL-induced pro-apoptotic crosstalk between the TRAIL-receptor pathway and TrkAIII involved SHP, c-Src and TrkAIII activity. TrkA (GW441756), c-Src (PP1) and SHP1/2 (NSC-87877) inhibitors significantly reduced TRAIL-induced TrkAIII SH-SY5Y apoptosis, which was associated with time-dependent changes in c-Src and TrkAIII phosphorylation. These changes were characterised by initial TRAIL-induced de-phosphorylation of c-Src inhibitory tyrosine Y527 combined with increased phosphorylation of c-Src active site tyrosine Y416, consistent with activation [63], which was accompanied by the induction of complexing between phosphorylated TrkAIII, SHP-1 and c-Src, followed by TrkAIII Y674/675 de-phosphorylation, consistent with inactivation [64], implicating SHP-1 and c-Src in TRAIL-induced pro-apoptotic crosstalk with TrkAIII. It remains unclear, however, whether both SHP-1 and c-Src, which directly bind activated TrkA receptors [65, 66], interact directly with TrkAIII under these conditions.

The SHP inhibitor NSC-87877 prevented both TRAIL-induced Src Y527 de-phosphorylation and increased Y416 phosphorylation. The c-Src inhibitor PP1 prevented TRAIL-induced c-Src Y416 phosphorylation but not Y527 de-phosphorylation, and both inhibitors prevented TRAIL-induced TrkAIII Y674/675 de-phosphorylation. This suggest that TRAIL first activates SHP, which de-phosphorylates c-Src Y527 within constitutive complexes leading to c-Src activation and Y416 auto phosphorylation, which in turn promotes interaction with phosphorylated TrkAIII, resulting in SHP-mediated TrkAIII Y674/675 de-phosphorylation and subsequent apoptosis. This possibility is supported by reports that SHP is recruited and activated at death receptors [67], TRAIL activates c-Src [68], c-Src and SHP bind activated TrkA receptors [65, 66] and SHP de-phosphorylates TrkA Y674 and Y675 residues [66].

The association between TRAIL-induced TrkAIII de-phosphorylation and apoptosis was confirmed by detection of TrkAIII Y674/675 phosphorylation in TRAIL-treated surviving but not apoptotic cells. TrkAIII de-phosphorylation alone, however, would appear to be insufficient to sensitize SH-SY5Y cells to TRAIL-induced apoptosis, since TrkAIII inactivated by GW441756 (this study and 8) significantly inhibited TRAIL-induced TrkAIII SH-SY5Y apoptosis. This suggests that a more complex interaction between c-Src/SHP and phosphorylated TrkAIII, resulting in TrkAIII de-phosphorylation is required for a pro-apoptotic outcome. Furthermore, a pro-apoptotic outcome may also reflect changes in the c-Src/SHP equilibrium, since activated c-Src promotes apoptotic resistance and TrkA phosphorylation [65, 68], whereas activated SHP promotes death receptor-mediated apoptosis and TrkA de-phosphorylation [66, 67], and both exhibit reciprocal regulation [69]. However, since inhibitors of both SHP and c-Src reduced TRAIL-induced TrkAIII SH-SY5Y apoptosis in association with maintenance of TrkAIII phosphorylation, both SHP and c-Src are required for TRAIL-induced pro-apoptotic cross talk between the TRAIL receptor pathway and TrkAIII.

In a direct comparison between TRAIL-treated apoptotic and surviving TrkAIII SH-SY5Y cells, increased complexing between cFLIP and de-phosphorylated TrkAIII was detected in apoptotic but not in surviving TrkAIII SH-SY5Y cells. This suggests that TRAIL may induce cFLIP sequester by de-phosphorylated TrkAIII with intracellular membranes [1, 8], reducing its recruitment to activated cell surface TRAIL receptors, which was confirmed by the time-dependent increase in caspase-8 to c-FLIP ratio recruited to DR4 positive receptors in TRAIL-treated TrkAIII SH-SY5Y but not NT-SH-SY5Y cells. This extend our previous reports that c-FLIP complexes with NGF-activated TrkA receptors [49, 70], sensitizing TrkA SH-SY5Y cells to TRAIL-induced apoptosis [49] to include a role for TrkAIII/cFLIP complexing in TRAIL-induced TrkAIII SH-SY5Y apoptosis. cFLIP, therefore, can interact with both phosphorylated TrkA and non-phosphorylated TrkAIII receptors depending upon the circumstance, which may reflect not only differences in receptor structure but also localisation, post receptor signaling and/or complex composition.

SiRNA Mcl-1 knockdown accelerated and augmented TRAIL-induced TrkAIII SH-SY5Y apoptosis, characterising Mcl-1 as a major regulator of TrkAIII SH-SY5Y sensitivity to TRAIL-induced apoptosis. In contrast to siRNA cFLIP knockdown, which accelerated and augmented TRAIL-induced apoptosis through the extrinsic pathway, z-LEHD-fmk caspase-9 inhibitor prevented accelerated and augmented TRAIL-induced apoptosis following siRNA Mcl-1 knockdown, confirming intrinsic apoptosis pathway involvement [23–25]. Interestingly, Mcl-1 expression in TrkAIII SH-SY5Y cells was inhibited by the PERK inhibitor GSK2656157 [71] but not by the TrkA inhibitor GW441756, whereas Bcl2 and Bcl-xL expression was reduced by GW441756 but not GSK2656157, implicating TrkAIII activity in Bcl-2 and Bcl-xL but not Mcl-1 expression and the PERK arm of the ER-stress response in the expression of Mcl-1 but not Bcl-2 and Bcl-xL. This supports reports of ER-stress regulated Mcl-1 expression [72] and the activation an ER-stress response survival adaptation in TrkAIII SH-SY5Y cells [11, 12], adding PERK up-regulated expression of Mcl-1 as a novel potential “non-oncogene addiction” mechanism to oncogenic TrkAIII repertoire.

Constitutive inhibitory caspase 8 Y380-phosphorylation [73] was detected at similar levels in all SH-SY5Y cell lines, providing an additional mechanism for reducing sensitivity to TRAIL-induced apoptosis. However, neither GW441756 nor TRAIL reduced caspase 8 Y380-phosphorylation in TrkAIII SH-SY5Y cells, indicating that caspase 8 Y380-phosphorylation does not depend upon TrkAIII and its de-phosphorylation was not involved in sensitizing TrkAIII SH-SY5Y cells to TRAIL-induced apoptosis.

In conclusion, we have identified a potential therapeutic “Achilles heel” for the TrkAIII oncoprotein, characterised by TRAIL-induced one-way pro-apoptotic crosstalk between the TRAIL receptor signaling pathway and TrkAIII, in SH-SY5Y NB cells. We propose that TRAIL-induced apoptosis initiates with TRAIL activation of SHP, followed by SHP-mediated activation of c-Src. This promotes complexing between phosphorylated TrkAIII, SHP and c-Src, leading to SHP-mediated TrkAIII de-phosphorylation and the sequester cFLIP, which increases the caspase-8 to cFLIP ratio recruited to activated TRAIL-receptors, explaining the delay in caspase cleavage and apoptosis via the extrinsic pathway. Our observations add significantly to a previous report of pro-apoptotic cross talk between TNF-α signaling networks and tyrosine kinase receptors [74] and provide a novel rational for the therapeutic use of TRAIL, combined with siRNA cFLIP and Mcl-1 inhibitors, in TrkAIII expressing NBs. Such an approach may work best in first-line therapy, as TRAIL promotes KRAS-driven metastasis [75] and KRAS mutations are detected in relapsed but not primary NBs [76–78].

## MATERIALS AND METHODS

### Cell lines and reagents

Non-transfected, pcDNA control transfected and TrkAIII transfected SH-SY5Y NB cell lines have been described previously [1]. Cells were grown in RPMI, supplemented with appropriate antibiotics (Zeocin for stable transfectants, penicillin and streptomycin) and 10% foetal calf serum. GW441756 TrkA inhibitor [51], z-VAD-fmk pan-caspase inhibitor, z-IETD-fmk caspase 8 inhibitor and z-LEHD-fmk caspase 9 inhibitor; human recombinant TRAIL and PP1 c-Src inhibitor were purchased from Sigma-Aldrich (St. Louis Mo). NSC-87877 SHP1/2 inhibitor and GSK2656157 PERK inhibitor were from MERCK/Millipore (Darmstadt, GE). Antibodies against TrkA, α-tubulin, c-FLIP, caspase-8, caspase-9, BID and Mcl-1 were purchased from SantaCruz (SantaCruz, Ca). Antibodies against Y674/675 phosphorylated TrkA, c-Src, Y527 phosphorylated and Y416 phosphorylated c-Src and Caspase-3, were from Cell Signalling (Danvers, MA). Antibodies against DR4, DR5, DcR1 and DcR2 were from ANASPEC (Belgium). Antibodies against Bcl2 and Bcl-xL were from AbCam (Cambridge, UK). Hybond C-extra nitrocellulose membranes and ECL solutions were purchased from Amersham International (Bedford, UK). RNA-easy RNA purification kits were purchased from Qiagen (Hilden, Ge). Mammalian pcDNA expression vectors for Bcl-xL and Flag-tagged c-FLIP were kindly provided by Dr. Francesca Zazzeroni (Univ. of L'Aquila) and have been described previously [79].

### Transient Bcl-xL and cFLIP expression

Bcl-xL and flag-tagged cFLIP were overexpressed in TrkAIII SH-SY5Y cells by transient transfection of mammalian expression vectors bearing Bcl-xL, FLAG-tagged cFLIP [79] or empty pcDNA. Briefly, 0.6 μg/ml of each vector were transfected into sub-confluent cell cultures using FUGENE transfection reagent as directed by the manufacturer (Promega, Madison, Wi). At 6 hours transfection medium was replaced with fresh growth medium (RPMI/10%FCS/glutamine plus antibiotic) and the cells left for 48 hours, at which time they were utilised for experimentation. Overexpression of Bcl-xL and Flag-tagged cFLIP was verified by Western blotting of total cell extracts (20 μg).

### SiRNA knockdown of c-FLIP and Mcl-1

C-FLIP and Mcl-1 expression was knocked down in TrkAIII SH-SY5Y cells using a TriFECTa Dicer-Substrate RNAi kit, employing three cFLIP-specific or three Mcl-1-specific Dicer-Substrate siRNA duplexes, as described by the manufacturer (Integrated DNA Technologies, Coralville, IA). Briefly, cells were subjected to 48 hour transient transfection with either 25 nM negative control siRNA duplex (provided with the kit) or 25 nM of a mix of cFLIP specific or Mcl-1 specific siRNA duplexes, using INTERFERin *in vitro* siRNA transfection reagent, as described by the manufacturer (Polyplus Transfection Inc., New York, NY). Sham transfected controls received transfection reagent alone. SiRNA knockdown of cFLIP and Mcl-1 expression was confirmed by Western blot comparison to α-tubulin in whole cell extracts (20μg).

### Mcl-1 siRNA sets

5′-AGCCUAGUAUGUCAAUAAAGCAAAT-3′ and 5′-AUUUGCUUUUAUUGACAUACUA GGCUUA-3′; 5′-GGAACAAAUCUGAUAACUAUGCAGG-3′ and 5′- CCUGCAUAGUUAUCAGAUUUGUUCCAC-3′; and 5′-CAAGUGCAUAGAUGUGAAUUGGUTT-3′ and 5′-AAACCAAUUCACAUCUAUGCACUUGUU-3′.

### cFLIP siRNA sets

5′-UGAGUUGGAGAAACUAAAUCUGGTT-3′ and 5′-AACCAGAUUUAGUUUCUCCAAC UCAAC-3′; 5′-CG AAGACCCUUGUGAGCUUCCCUAG-3′ and 5′-CUAG GGAAGCUCACA AGGGUCUUGCAG-3′; and 5′-GCC GAGGCAAGAUAAGCAAGGAGAA-3′ and 5′-UUC UCCUUGCUUAUCUUGCCUCGGCCC-3′

Transfection efficiency was confirmed using a HPRT-S1 DS positive control and validated using a negative control duplex (NC1) not present in the human genome, as described by the manufacturer (Integrated DNA Technologies; www.IDTDNA.com)

### Cell extraction, immunoprecipitation and Western blotting

Cells were extracted in lysis buffer (PBS containing 0.5% sodium deoxycholate, 1% NP40, 0.1% SDS, 1 mM sodium orthovanadate, 1 mM PMSF, 1 μg/ml of pepstatin A and Aprotinin) and protein concentrations were calculated by Bradford protein concentration assay (Sigma-Aldrich). Samples were mixed with reducing SDS-PAGE sample buffer and subjected to reducing SDS-PAGE/Western blotting. Briefly, proteins separated by reducing SDS-PAGE, were trans-blotted onto Hybond C+ nitrocellulose membranes by electrophoresis (Amersham Int. UK) and the membranes subsequently air-dried. Non-specific protein binding-site on membranes were blocked by incubation for 2 hours in 5% non-fat milk in TBS-T prior to incubation with primary antibodies, at recommended dilutions, for 2–16 hours at 4°C. Membranes were then washed in TBS-T, incubated with secondary HRP-conjugated antibodies (Jackson ImmunoResearch Laboratories, West Grove, PA) diluted in blocking solution and immunoreactive species detected by chemiluminescence reaction, as directed by the manufacturer (Amersham Int).

### RNA purification and RT-PCR

RT reactions were performed on total RNAs (1 μg), purified using RNA-easy Plus, as described by the manufacturer (Qiagen), using the Moloney Murine Leukemia virus RT kit, as detailed by the manufacturer (LifeTechnologies, Inc, Paisley, UK). RT reactions were subjected to PCR using the following primers:

GAP: 5′- AGGTCCACCACTGACAGTT-3′ (forward) and 5′- CTGCACCACCAACTGCTT AG-3′ (reverse) (300 bp).

DcR1: 5′-GAAGAATTTGGTGCCAATGCCACTG-3′ (forward) and 5′- CTCTTGGACTTGGCTGGGAGA TGTG-3′ (reverse) (612bp);

DcR2: 5′-CCCCCGGCAGGACGAAGTT-3′ (forward) and 5′- CTCCTCCGCTGCTGGGGTTTT-3′ (reverse) (418 bp);

DR4: 5′-ACTTTGGTTGTTCCGTTGCTGTTG-3′ (forward) and 5′-GGCTTTCCATTTGCTGCTCA-3′ (reverse) (214 bp);

DR5: 5′-CTGAAAGGCATCTGCTCAGGTG-3′ (forward) and 5′-CAGAGTCTGCATTACCTTCTAG-3′ (reverse) (347 bp);

c-FLIPs: 5′-GGACCTTGTGGTTGAGTTGG-3′ (forward) and 5′-ATCAGGACAATGGGCATAGG-3′ (reverse) (241 bp);

c-FLIP_L_: 5′-GGCTCCCAGAGTGTGTATGG-3′ (forward) and 5′-AGCTTCTCGGTGAACTGTGC-3′ (reverse) (249 bp);

Bcl-2: 5′-GACTTCGCCGAGATGTCC-3′ (forward) and 5′-CAAGCTCCCACCAGGGCCAAAC-3′ (reverse) (356 bp);

Bcl-XL: 5′-GTGAATTCTGAGGCCAAGGGAAC-3′ (forward) and 5′-GAACGGCGGCTGGGATACTTTTG-3′ (reverse) (373 bp);

Caspase-8: 5′-TCTGGAGCATCTGCTGTCTG-3′ (forward) and 5′-CCTGCCTGGTGTCTGAAGTT-3′ (reverse) (427 bp);

Caspase-10: 5′-GGGAACGGACACACAACTCT-3′ (forward) and 5′-CTAGCTTTTGGCCCTGACTG-3′ (reverse) (293 bp);

Caspase-3: 5′-TTAATAAAGGTATCCATGGAGAACACT-3′ (forward) and 5′-TTAGTGATAAAAATAGAGTTCT TTTGTGAG-3′ (reverse) (849 bp);

Mcl-1: 5′-AAGCC AATGGGCAGGTCT-3′ (forward) and 5′-TGTCCAGTTTCCGAAGCAT-3′ (reverse) (121 bp);

For each primer set, PCRs were performed on reverse transcription reactions serially diluted from 1 to 1:1000. Reactions below saturation were compared by densitometric analysis of Jpeg images of ethidium bromide stained gels, using ImageJ64 software [80].

### Cell death assay

TRAIL-induced cell death was routinely assayed using a modification of previously described methods [81, 82]. Briefly, cells were washed once in Ca^2+^ free PBS, detached with ice cold PBS containing 1 mM EDTA, transferred to sterile 15 mls tubes, centrifuged for 5 minutes at 1,000 × g at 4°C, washed with ice cold PBS and re-pelleted by centrifugation at 1,000 × g for 5 minutes, at 4°C. Cell pellets were re-suspended in 25 μl of PBS containing 2 μl of acridine orange/ethidium bromide solution (100 μg/ml acradine orange and 100 μg/ml ethidium bromide in PBS) plated onto glass slides and examined immediately under a Zeiss “Axioplan-2” fluorescence microscope. Representative fields were digitally photographed under identical exposure conditions and the number of dead cells (orange/red nuclei) and live cells (green nuclei) counted. In addition, phase contrast micrographs of parallel cultures were used to confirm changes in the relative percentage of adherent (surviving) and suspension (apoptotic) cells, following TRAIL-treatment. TRAIL-induced TrkAIII SH-SY5Y cell apoptosis was confirmed by its complete inhibition by z-VAD-fmk pan caspase inhibitor.

### Tumour growth in soft agar

For substrate-independent tumour growth assays [49], 1 × 10^4^ cells in single-cell suspension (passed through a gauge × 18 syringe needle) were mixed in a 33% solution of agar (BiTec; Difco) in RPMI containing 5% FCS at 37°C, with or without TRAIL (200 ng/ml) and layered onto a solid 0.6% agarose substrate also with or without TRAIL, prepared in the same growth medium. Following top phase agar solidification, complete medium was added and re-placed every 2 days. Tumour spheroid growth was monitored over a 14-day period by phase contrast microscopy. Tumour spheroids were counted in 10 random fields at ×10 magnification.

### Isolation of TRAIL-activated death receptor complexes

TRAIL-ligated death receptor complexes were purified from biotin-TRAIL treated pcDNA SH-SY5Y and TrkAIII SH-SY5Y cells by ligand affinity precipitation, as previously described [83]. Briefly, biotinylated TRAIL was prepared by incubating TRAIL (1 mg/ml) with Sulfo-NHS-LC-Biotin (1 mg/ml) (Pierce) for 1 hour on ice. The reaction was stopped by adding 1/10 volume of 1M Tris-HCl [pH. 7.5] and unincorporated biotin removed by buffer exchange into 150 mM NaCl, 30 mM HEPES (pH 7.5) using PD-10 columns (Amersham Pharmacia Biotech). For ligand affinity precipitation, cells (5 × 10^6^ cells per sample) were washed twice in RPMI at 37°C and incubated with 1 mg/ml biotinylated TRAIL for 1 and 6 hours. Death receptor complex formation was stopped by the addition of 15 volumes of ice cold PBS and cells were then lysed in 4.5 mls of lysis buffer (30 mM TRIS-HCl [pH. 7.5], 150 mM NaCl, 10% glycerol, 1% Triton X-100, supplemented with complete protease inhibitor cocktail) (Roche Diagnostics, Mannheim, GE). TRAIL receptor protein complexes were precipitated from lysates by co-incubation with 20 μl of Streptavidin beads (Pierce) for 3 hours at 4°C with rotation. Ligand affinity precipitates were washed four times in lysis buffer, eluted from beads in reducing SDS-PAGE sample buffer and subjected to SDS-PAGE Western blotting.

### Indirect immunofluorescence

Cells grown on Nunc glass chamber slides (Sigma-Aldrich) were washed in PBS, fixed in 4% paraformaldehyde, washed in PBS then processed for indirect immunofluorescence (IF). Fixed, non-permeabilised cells were incubated for 1 h in blocking solution (1% bovine serum albumin in PBS-0.03% TX100) and then incubated for 2 hours with primary antibody in blocking solution at room temperature. Slides were washed three times in PBS-0.03% TX100, incubated with secondary fluorochrome-conjugated antibody diluted in blocking solution for 1 h at room temperature, washed in PBS-0.03% TX100 and mounted using VectorMount. IF images were obtained using a Zeiss Axioplan 2 fluorescence microscope with a digital camera and Leica M500 Image Manager software.

### Statistical analysis

Data were analysed statistically by Student's *t-test* and statistical significance was associated with probabilities of < 0.05.
